# Caspase-9 suppresses metastatic behavior of MDA-MB-231 cells in an adaptive organoid model

**DOI:** 10.1038/s41598-024-65711-z

**Published:** 2024-07-02

**Authors:** Farzaneh Falahi, Shiva Akbari-Birgani, Yousef Mortazavi, Behrooz Johari

**Affiliations:** 1 Department of Medical Biotechnology, School of Medicine, Zanjan University of Medical Sciences, Zanjan, Iran; 2 Cancer Gene Therapy Research Center, Zanjan University of Medical Sciences, Zanjan, Iran; 3 Department of Biological Sciences, Institute for Advanced Studies in Basic Sciences (IASBS), Zanjan, 45137-66731 Iran; 4 Research Center for Basic Sciences and Modern Technologies (RBST), Institute for Advanced Studies in Basic Sciences (IASBS), Zanjan, 45137-66731 Iran

**Keywords:** Triple-negative breast cancer (TNBC), Metastasis, Inducible caspase-9, Organotypic model, Three-dimensional cell culture, Biochemistry, Biotechnology, Cancer, Cell biology

## Abstract

Caspase-9, a cysteine-aspartate protease traditionally associated with intrinsic apoptosis, has recently emerged as having non-apoptotic roles, including influencing cell migration—an aspect that has received limited attention in existing studies. In our investigation, we aimed to explore the impact of caspase-9 on the migration and invasion behaviors of MDA-MB-231, a triple-negative breast cancer (TNBC) cell line known for its metastatic properties. We established a stable cell line expressing an inducible caspase-9 (iC9) in MDA-MB-231 and assessed their metastatic behavior using both monolayer and the 3D organotypic model in co-culture with human Foreskin fibroblasts (HFF). Our findings revealed that caspase-9 had an inhibitory effect on migration and invasion in both models. In monolayer culture, caspase-9 effectively suppressed the migration and invasion of MDA-MB-231 cells, comparable to the anti-metastatic agent panitumumab (Pan). Notably, the combination of caspase-9 and Pan exhibited a significant additional effect in reducing metastatic behavior. Interestingly, caspase-9 demonstrated superior efficacy compared to Pan in the organotypic model. Molecular analysis showed down regulation of epithelial–mesenchymal transition and migratory markers, in caspase-9 activated cells. Additionally, flow cytometry analysis indicated a cell cycle arrest. Moreover, pre-treatment with activated caspase-9 sensitized cells to the chemotherapy of doxorubicin, thereby enhancing its effectiveness. In conclusion, the anti-metastatic potential of caspase-9 presents avenues for the development of novel therapeutic approaches for TNBC/metastatic breast cancer. Although more studies need to figure out the exact involving mechanisms behind this behavior.

## Introduction

Breast cancer (BC) stands out as the most frequently diagnosed cancer in women and is a leading cause of cancer-related fatalities worldwide. According to data from the American Society, it is projected that in 2023, breast cancer will account for 30,590 new cases (29,770 in women, 2800 in men) and 43,700 deaths (43,170 in women, 530 in men)^1,2^. BC is classified into subtypes based on estrogen/progesterone receptors (ER/PR), HER2, and Ki67^3,4^. Triple-negative breast cancer (TNBC) is a particularly aggressive subtype of BC that is characterized by the absence of estrogen/progesterone receptor, and HER2 expression, constituting 15–20% of all breast cancers. Concerning, approximately 45% of TNBC patients experience distant metastases, often affecting the bones or other parts of the body, leading to a reduction in median survival from 13.3 months to 18 months^5–7^. Despite recent progress in therapies and treatment strategies, the lack of a specific molecular target or receptor hampers efforts to effectively address TNBC. While traditional chemotherapy has shown some efficacy in TNBC patients, its toxic effects pose risks to patients, and a subset fails to derive any clinical benefits. Consequently, identifying effective targets for precise TNBC therapy remains a challenging and crucial clinical issue that requires attention^8–10^.

In the realm of cancer research, the well-established and extensively studied monolayer cell culture has played a crucial role in advancing our understanding of carcinogenesis. It has provided insights into various facets, ranging from cell proliferation and migration to the discovery of new drugs. However, cancer, being a highly diverse disease, involves a complex and dynamic tumor microenvironment (TME). This environment includes various cellular components such as stem cells, tumor epithelium, fibroblasts, and endothelial cells, along with non-cellular elements like cytokines, chemokine, extracellular matrix (ECM), and growth factors. The interaction between tumor cells and their surrounding matrix, as well as the physical and biochemical properties of this microenvironment, plays a key role in regulating cancer differentiation, proliferation, invasion, and metastasis^11,12^. Nevertheless, monolayer cell cultures fall short in replicating the intricate and dynamic interactions present in the TME, especially spatial cell–ECM and cell–cell interactions. Multicellular three-dimensional (3D) organoids represent a 3D in vitro model system that offers more physiologically relevant tumor models compared to conventional two-dimensional (2D) cultures. By capturing cell-to-cell and cell-to-matrix interactions, cell polarization, nutrient, and gas diffusion gradients, as well as metabolic processes, these models more accurately reflect the complexity and interactions observed in real tumors. As a result, they are more suitable for studying metastasis, invasion, and the screening of therapeutic drugs^12–15^.

Caspases are cysteine-aspartate proteases primarily recognized as effectors of apoptosis^16^. Recent studies have revealed that caspases play various non-apoptotic roles, such as influencing cell identity during differentiation and dedifferentiation, reconstructing the cytoskeleton, impacting cell growth and the cell cycle, and interfering with immune responses^17,18^.

Caspase-9 serves as an initiator caspase, triggering the internal pathway of apoptosis. Upon its activation, executive caspase-3/7 are also activated. However, recent research has demonstrated that non-lethal activation of caspase-9 can lead to non-apoptotic roles, affecting cell homeostasis, autophagy, differentiation, and activation of NF-KB pro-survival pathways^17–19^. For instance, inhibiting caspase-9 activity can disrupt the differentiation of muscle and nerve cells^20,21^. The relationship between caspases and cell migration has been a recent topic of discussion. Migration involves the restoration of cell dynamics without altering its contents. Studies have indicated that reducing caspase-3 expression can diminish the migration of colorectal cancer cells in vitro and their metastasis in xenograft models^22^. On the contrary, recent research conducted in Drosophila indicates that the sub-lethal activity of (Dronc and Drice, analogs of human caspase-9 and caspase-3 respectively) may potentially diminish cell migration and invasion^23,24^.

The inducible caspase9 is a genetically modified form of human caspase 9, notable for its absence of Caspase Activation and Recruitment Domain (CARD). Consequently, its dimerization and activation require a chemical inducer of dimerization such as AP20187 or AP1903 that their safety on human cells were proven^25,26^.

According to these findings, we conducted a study to investigate the impact of caspase-9 activation using the inducible caspase-9 on the metastatic behavior of MDA-MB-231 cells in both 2D and an organotypic model derived from MDA-MB231 and Fibroblast cells and Panitumumab was employed as a reference anti-metastatic agent.

## Results

### Caspas-9 expression associates with clinical outcome of breast cancer patients

**Figure 1:**
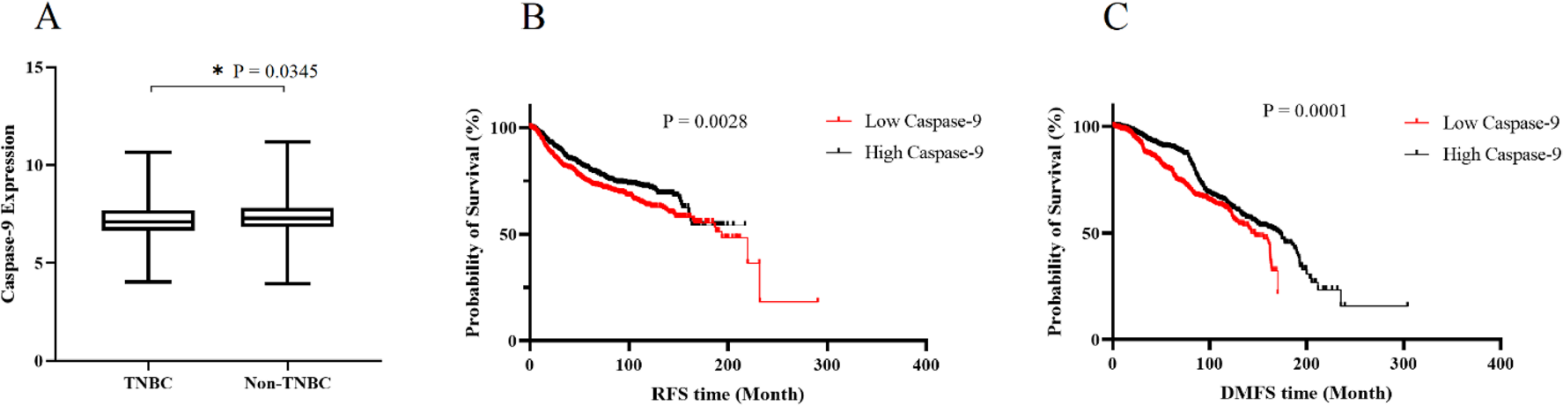
Investigation of caspase-9 expression in breast cancer patients. (**A**) Differential expression of caspase-9 in 1337 TNBC and 5656 non-TNBC patients. (**B**) RFS analysis were performed in 1665 breast cancer patients; 917 and 748 patients with low and high expression of caspase-9, respectively. (**C**) DMFS analysis were performed in 2619 breast cancer patients; 1368 and 1251 patients with low and high expression of caspase-9, respectively. Significant differences are denoted as; *p < 0.05, **p < 0.01, and ****p < 0.0001.

As a prelude to testing the possible association between caspase-9 with metastasis in breast cancer, the expression of caspase-9 in TNBC and non-TNBC tumors was investigated in the stratified breast cancer patients from BCIP database. As shown in Fig. 1A, caspase-9 is significantly less expressed in TNBC patients than in non-TNBC patients. Furthermore, to evaluate the association of caspase-9 expression with metastasis and disease recurrence, relapse-free survival (RFS) and distant metastasis-free survival (DMFS) were evaluated, too. As shown in Fig. 1B, RFS analysis for 917 patients with high expression of caspase-9 and 748 patients with low expression of caspase-9 shows that the lower expression of caspase-9 is significantly related to the shorter relapse free survival. Likely, DMFS comparison between 1368 patients with high expression of caspase-9 and 1251 patients with low expression of caspase 9 in Fig. 1C reveals that low expression of caspase-9 significantly associates with shorter distant metastasis-free survival.

### The iC9 exhibited expression and activation in the established iC9-transduced cell

**Figure 2:**
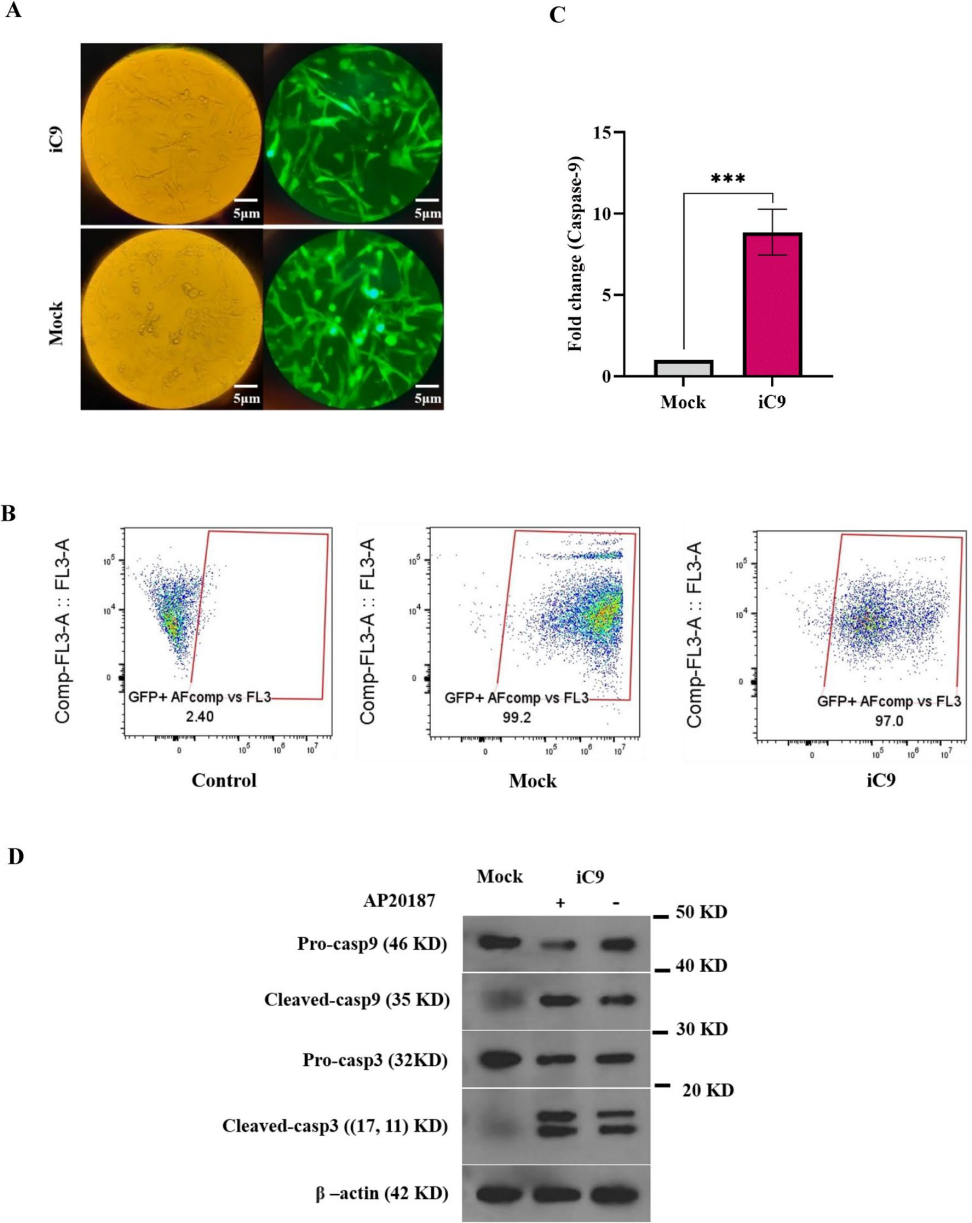
iC9 overexpressed in the MA-MB-231 stable cell line. (**A**) The efficiency of cell transduction was measured using fluorescent microscopy (Magnification; 40×, Scale bar; 5 µm). (**B**) The percentage of GFP-positive cells was determined through flow cytometry analysis. (**C**) The relative overexpression levels of iC9 in the iC9-transduced group, compared to the mock-transduced control, were determined using RT-PCR, GAPDH was considered as an internal control. The data is presented as the mean of fold change ± SD (N = 3), with significance denoted as ***p < 0.001. (**D**) The protein levels and activation of caspase-9 and caspase-3 in Mock or iC9-transduced cells with or without AP20187 treatment were assessed using immunoblotting.

To assess the transduction efficiency in stable cell lines one month after transduction and puromycin selection, the quality of GFP expression was evaluated using fluorescent microscopy and quantified via flow cytometry. As depicted in Fig. 2A, GFP was prominently expressed in both the mock and iC9-transduced groups. Furthermore, in Fig. 2B the flow cytometry quantification indicated 99.2% and 97% expression of this gene in the mock and iC9-transduced groups, respectively.

For a more comprehensive investigation, the up regulation of caspase-9 RNA expression was assessed using real-time PCR, while the protein expression and activation of caspase-9/3 were examined through western blotting. As illustrated in Fig. 2C Real-time PCR analysis revealed a significant 8.86-fold increase (p-value 0.0006) in caspase-9 expression in the iC9 group compared to the mock group.

Figure 2D displays that the amount of cleaved caspase-9 (active form) exhibited a remarkable increase in the iC9-transduced groups (1.41 in the non-treated group and 1.61 in the group treated with AP20187). Notably, a similar trend was observed in the cleaved forms of caspase-3, with a 1.77 and 2.34-fold increase in non-treated and treated iC9-transduced groups compared to the mock-transduced group. Consequently, these results confirm the overexpression and activation of iC9 in the established cell line.

### Caspase-9 activation in MDA-MB231 cells blocks the cell proliferation

**Figure 3:**
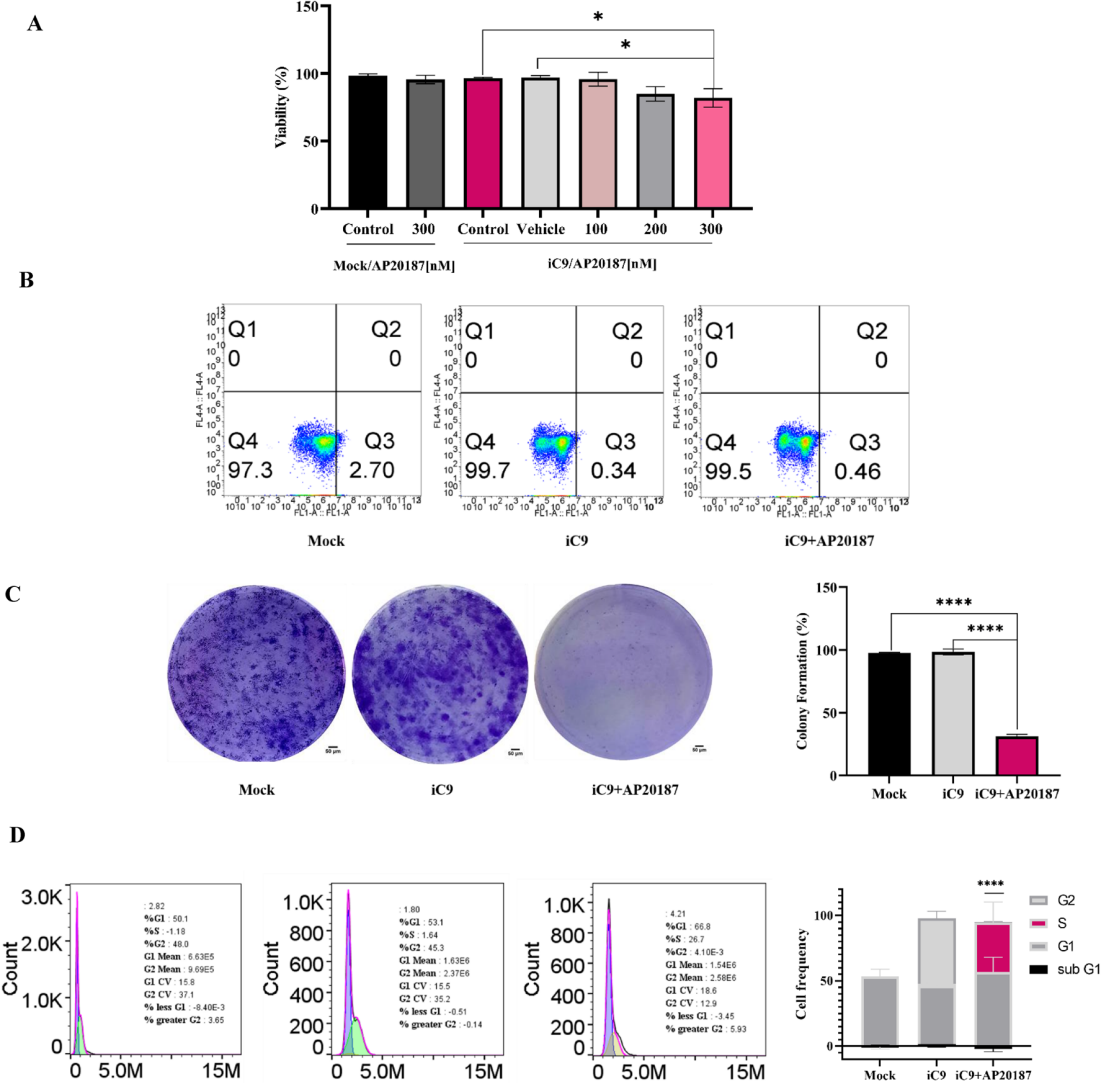
The cell viability and colony formation capacity of iC9-transduced cells decreased upon AP20187 treatment. (**A**) The percentage of viable cells after exposure to various concentrations (100–300 nM) of AP20187 was determined using the MTT assay. The vehicle was defined as cells treated with the maximum dose of DMSO present in 300 nm AP20187 (N = 3), with significance levels denoted as *p < 0.05. (**B**) The apoptosis analysis of mock-transduced cells and iC9-transduced cells with or without 300 nM AP20187 was measured by flow cytometry. (**C**) The percentage of colony formation by Mock or iC9-transduced cells with and without 300 nM AP20187 was also assessed (Scale Bar; 50 µm) (N = 2), with significance levels denoted as ****p < 0.001. (**D**) The cell cycle analysis of cells was done in all groups (N = 3), with significance levels denoted as ****p < 0.001.

The cytotoxic effect of iC9 on the MDA-MB-231 cell line was investigated, and it was observed that iC9-transduced cells treated with AP20187 did not show significant cytotoxicity. In Fig. 3A, the MTT assay, insignificant cell death was observed in the majority of the groups treated with increasing concentrations of AP20187 (100–300 nM). Only the 300 nM AP20187 administration demonstrated a notable (14.59%) decrease in absorbance compared to other concentrations leading to its selection for further experimentation in this study. Additionally, result revealed that the AP20187 administration had not any cytotoxicity effect on non iC9 transduced cells (mock). Furthermore, an apoptosis assay was performed using flow cytometry, as showed in Fig. 3B, the iC9/AP20187 could not induce apoptosis in MDA-MB-231 cells.

In next step, the impact of the iC9/AP20187 system on the colony formation of MDA-MB-231 cells was assessed. As illustrated in Fig. 3C, a notable variation in the frequency of colonies was observed in iC9-transduced cells treated with 300 nM AP20187 compared to the other groups.

To elucidate the cause of this deceleration, a cell cycle analysis was performed using flow cytometer, as delineated in Fig. 3D, the AP20187-treated cells were arrested in the S phase, unlike the mock and non–treated iC9-transduced cells. This finding suggests that iC9/AP20187 may not significantly affect the cell viability of MDA-MB-231 cells, but it could have a detrimental effect on their colony formation ability.

### Organotypic model mimics the pathological features of TNBC tumor

**Figure 4:**
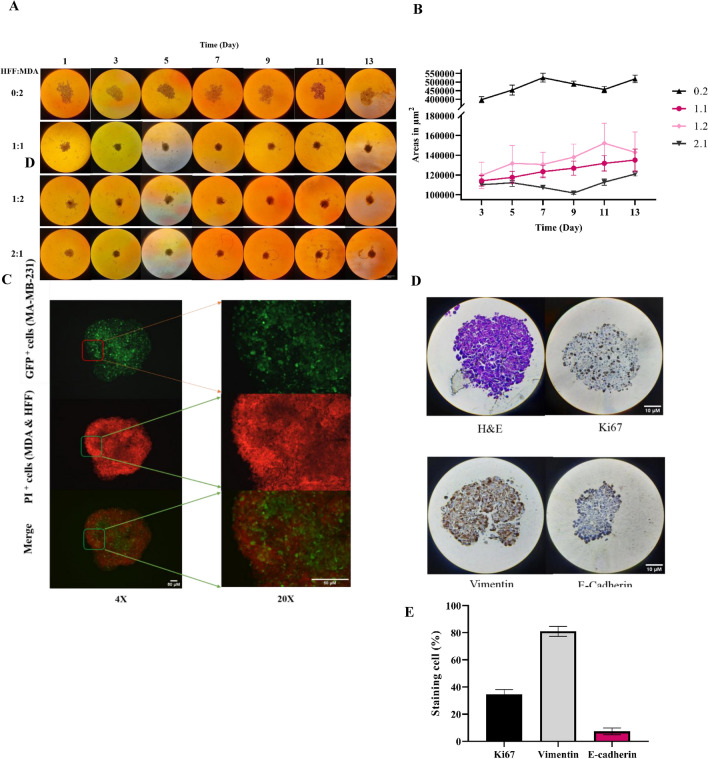
Generation of an organotypic model using different ratios of MDA-MB-231 and HFF cells and its characterization. (**A**) The size alternation of organoids generated from different ratios (0:2, 1:1, 1:2, 2:1) of HFF and MDA-MB-231 cells, was observed over 1 to 13 days. Images were taken every other day (Magnification 10×, Scale Bar is 300 µm). (**B**) Their growth kinetics were measured by following the changes in the size of organoids in a 13-day time course from day 3. The experiments were conducted in four replicates. (**C**) The cellular distribution was examined by fluorescent imaging. The GFP^+^ cells (MDA-MB-231) were visualized in green, while PI-stained cells (MDA and HFF) were depicted in red (Magnification 4× and 20×, Scale Bar is 50 µm) (**D**). The histopathological features of the organoids were studied by H&E and immunostaining of Ki67. (**E**) The mesenchymal feature of the organoids was investigated by immunostaining of Vimentin and E-cadherin (Magnification 40×, Scale bar is 10 μm). (**F**) The quantified data of (**D**) and (**E**).

To explore the impact of iC9/AP20187 in a more complex and physiologically relevant model, an organotypic model derived from MDA-MB-231 and human foreskin fibroblast (HFF) cells using a free scaffold 3D cell culture method was generated. The organoids comprised different ratios of MDA-MB-231 and HFF cells. Figure 4A illustrates that MDA-MB-231 cells alone were unable to form a well-defined 3D model, in contrast to the robust organotypic formation observed in co-culture with HFF cells. As shown in Fig. 4B, among the different ratios used for organotypic formation, the 1:1 ratio exhibited the longest maintenance in the logarithmic growth phase and the most suitable growth kinetics. Consequently, this ratio was selected and applied in further studies.

Figure 4C presents fluorescent microscopy images of the organoids in which all the nuclei of cells (MDA-MB-231 and HFF) are in red (stained with PI) and the MDA-MB-231 cells are in green (GFP expressing cells). The images clearly show the distribution of these two cell types within the organotypic model. H&E staining in Fig. 4D shows the typical feature of TNBC tumor tissue which mimicked by organoid models. Additionally, the IHC staining of Ki67 shows the proliferative cells and their heterogeneity within organotypic model. The high expression of Vimentin and low expression of E-cadherin in the organotypic model in Fig. 3D confirm the high mesenchymal and low epithelial characteristics of the cancer cells within the organoid. The stained cells were quantified in Fig. 4E.

### Caspase-9 activation alters the growth kinetics of the MDA-MB-231 cells in the organotypic model

**Figure 5:**
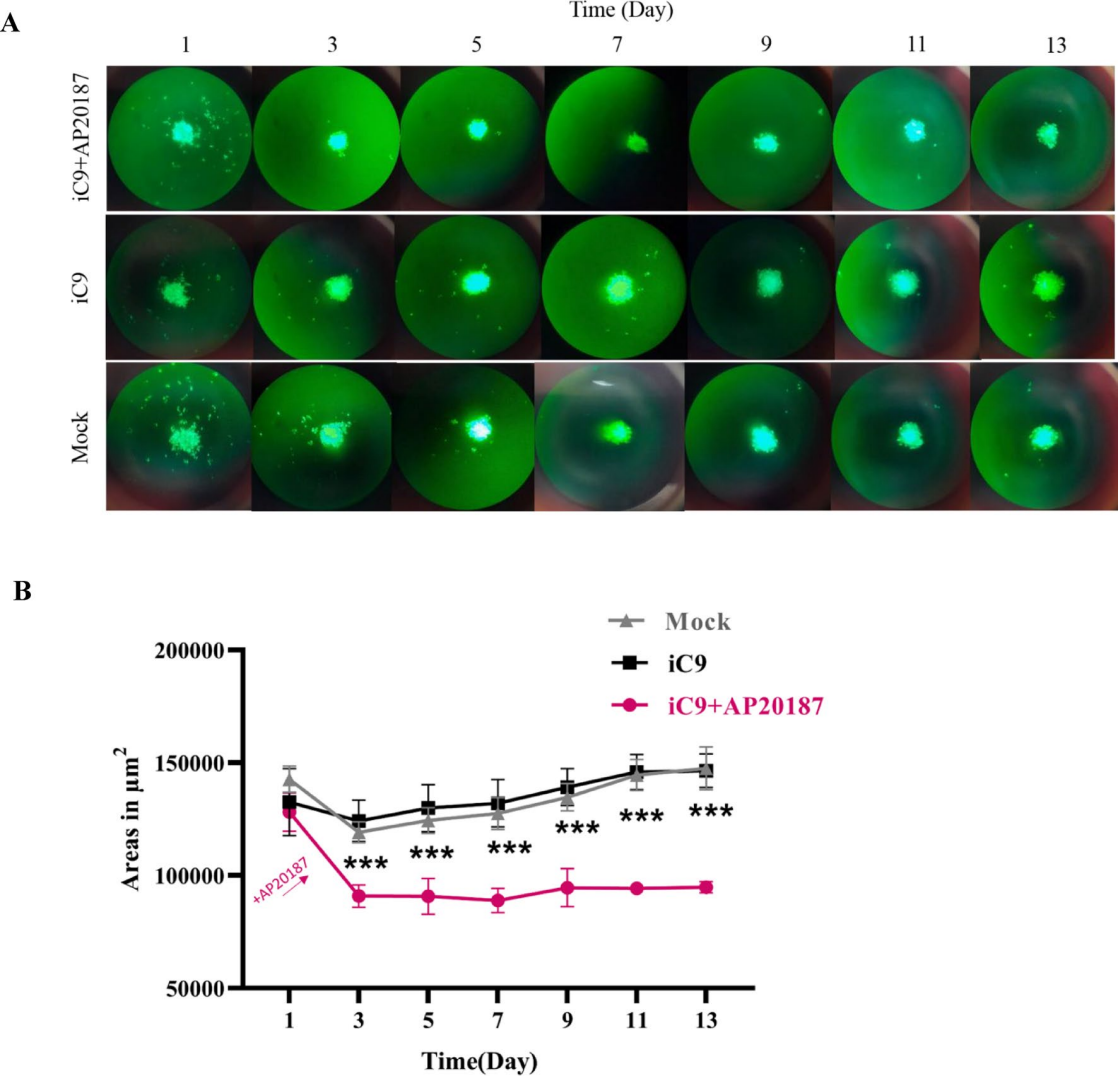
Growth kinetics of the organoids alters upon AP20187 treatment. (**A**) Organoids derived Mock-transduced cells and iC9-expressing cells, with or without AP20187 (300 nM) treatment, were observed over 1 to 13 days using fluorescent microscopy (Magnification 10×, Scale Bar is 50 µm). (**B**) The growth kinetics of organoids were measured by following the changes in the size of organoids over time. The experiments were conducted in three replicates and the significance levels are indicated as ***p < 0.001.

To evaluate the effect of AP20187 treatment on the growth characterization of iC9-transduced cells in an organismic model, a two-week growth kinetic analysis was conducted. Figure 5A depicts the fluorescent microscopy of organotypic models derived from mock or iC9-transduced cells with or without 300 nM AP20187 administration over 14 days, along with a graph in Fig. 5B quantifying their size alteration over time. The results indicate that organotypic models derived from iC9-transduced cells treated with 300 nM AP20187 exhibited a slower growth kinetic compared to non-treated models.

### Caspase-9 activation suppresses the migration and invasion of MDA-MB-231 cells

**Figure 6:**
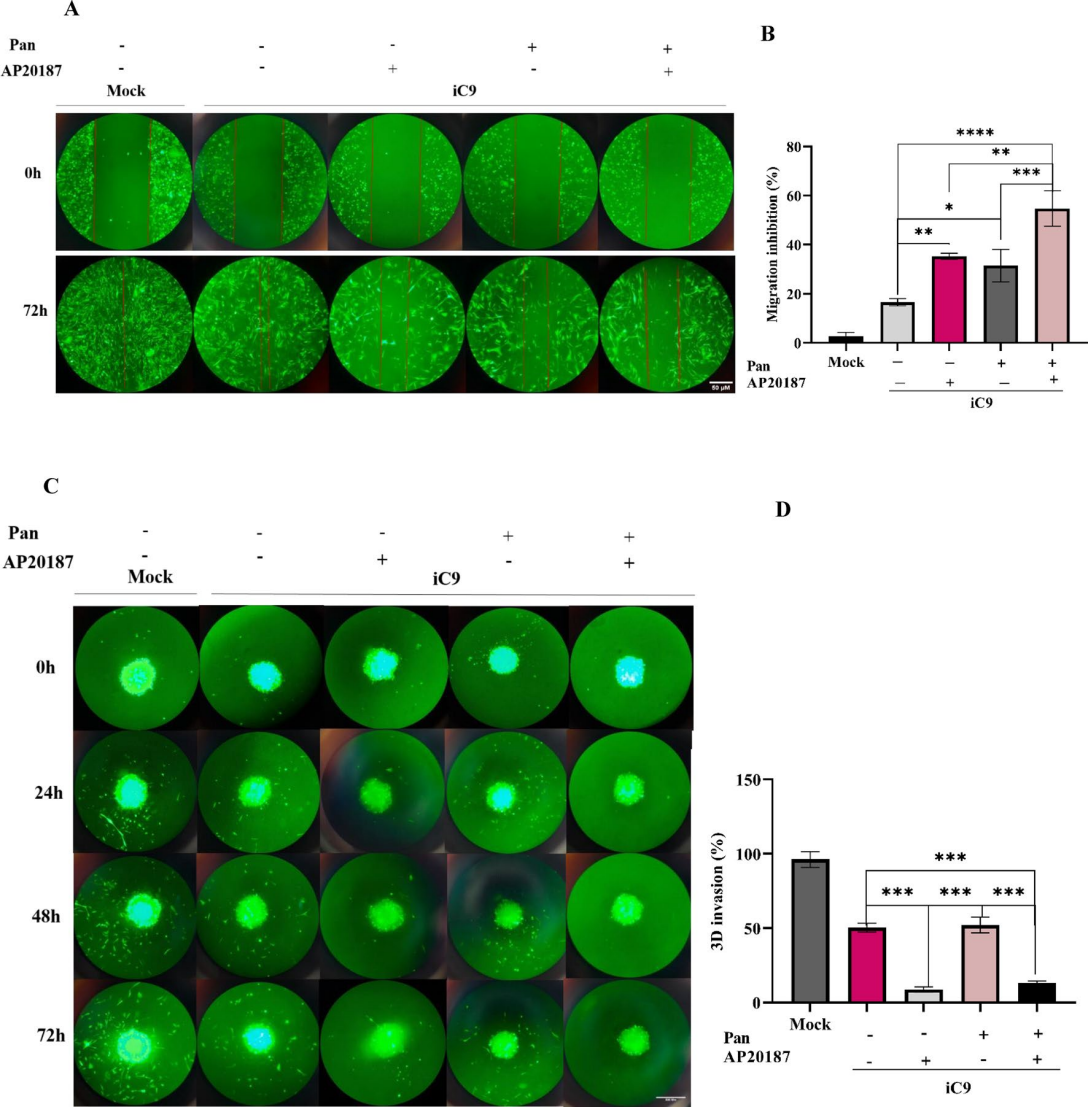
The iC9/AP20187 system reduces the migration and invasion of MDA-MB-231 cells. (**A**) To capture images of migrated cells in the monolayer under different media conditions with or without AP20187 and Pan, imaging was employed over a 72-h time course (magnification 10×, Scale Bar; 50 µm). (**B**) The migration inhibition percentage in wound healing assay was quantified. The data are expressed as mean ± SD of three independent replicates with significant differences denoted as *p < 0.05, **p < 0.01, and ***p < 0.001. The significant differences of mock group compare to iC9 group was (p-value = 0.025) and with other groups was less than 0.001. (**C**) A 3D invasion assay was conducted under similar media conditions over 3 days. The invaded cells from the organotypic model through a collagen matrix were imaged (Magnification 10×, Scale Bar is 50 µm). (**D**) And invasion was quantified. The Data are expressed as mean ± SD of three independent replicates with significant differences denoted as ***p < 0.001. The significant differences of mock group compare to other groups was less than 0.001.

To assess the influence of the iC9/AP20187 system on cell migration within a 2D microenvironment, a scratch assay was conducted. The result demonstrated that the migration rate of mock cells was higher than all iC9-transduced cells in all groups. Also, the results showed a significant increase (18.8%, p value 0.0034) in the inhibition of migration rate among AP20187 treated iC9-transduced cells compared to the untreated group. As illustrated in Fig. 6A,B, Pan Treatment also increased, albeit to a lesser extent (14.99%, p value 0.0155). Notably, the combination of AP20187 and Pan led to the most substantial increase in the inhibition of migration rate (38.27%, p value 0.0001).

Furthermore, the impact of the iC9/AP20187 system on the migration and invasion of MDA-MB-231 cells in an organotypic model was analyzed using a collagen matrix invasion assay. As depicted in Fig. 6C,D the migration and invasion capability of mock cells were higher than all iC9-transduced cells. Also result shows that AP20187 administration markedly reduces the migration and invasion rate in organotypic derived iC9-transduced cells from 50.4% in the non-treatment group to 8%, p value 0.0007. Interestingly, Pan Treatment did not significantly reduce the migration and invasion rate in organotypic derived iC9-transduced cells compared to other treatments. These findings underscore the significant role of caspase-9 in reducing migration and invasion in both 2D and 3D models, while Pan's ability to inhibit migration is more pronounced in the 2D model than the 3D model.

### Caspase-9 activation reduces EMT in MDA-MB-231 cells

In order to examine the impact of caspase-9 activation on the Epithelial–Mesenchymal Transition (EMT) of cells, an IHC analysis was performed on the organotypic model with and without AP20187 administration to assess the expression of Vimentin and E-Cadherin within these cells.

**Figure 7:**
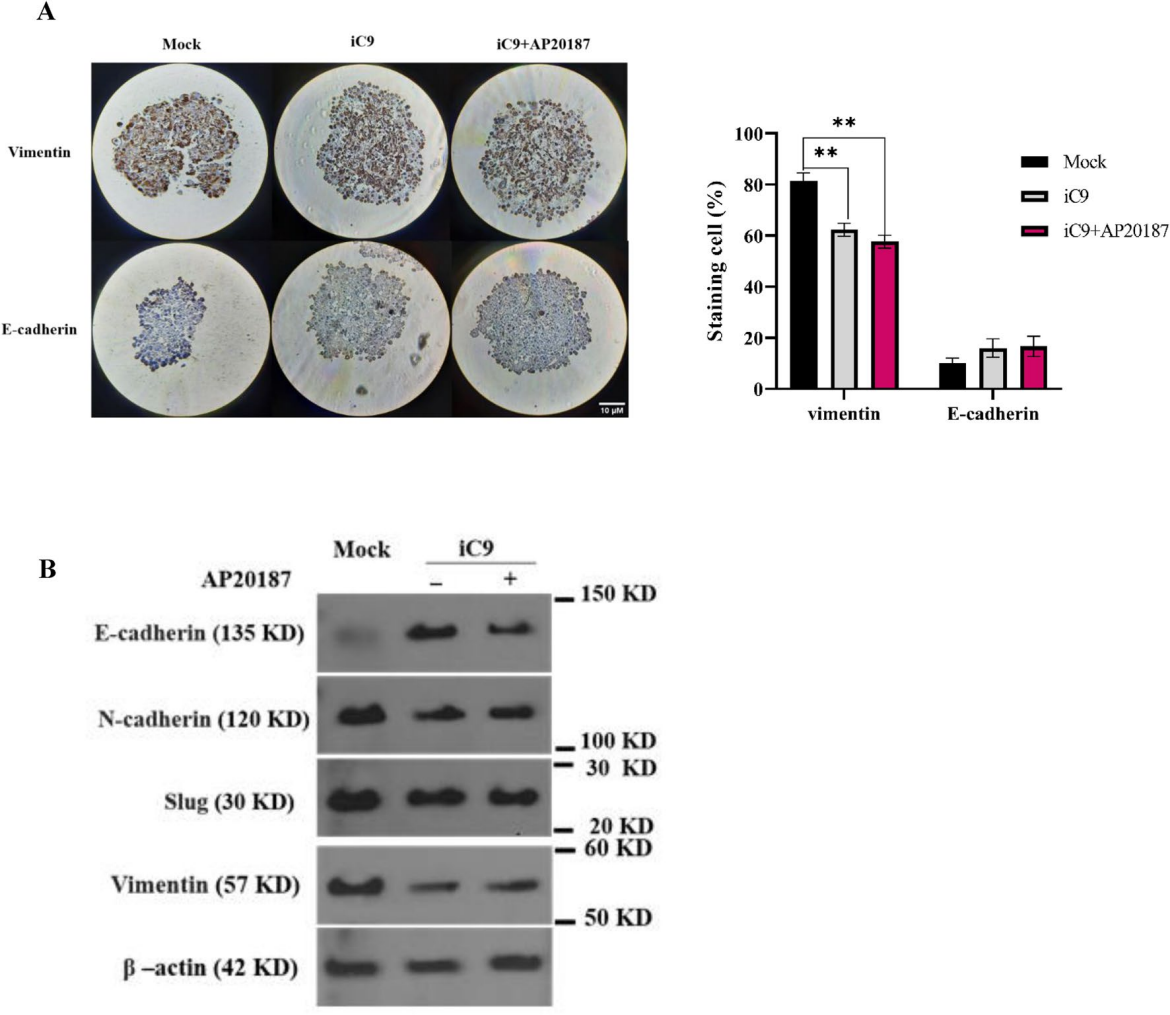
The iC9/AP20187 system reduces EMT in MDA-MB231 cells. (**A**) IHC staining of Vimentin and E-cadherin in the organotypic model was performed with or without 300 nM AP20187. The positively stained cells were visualized as brown color (Magnification 40×, Scale bar is 10 μm) and quantified. The data is presented for three replicates, and significance levels are illustrated as *p < 0.05 and ***p < 0.001. (**B**) The protein levels of EMT markers (E-cadherin, N-cadherin, Slug and Vimentin) in Mock, iC9-transduced cells with or without AP20187 treatment were assessed using immunoblotting. β-actin was considered as an internal control.

As depicted in Fig. 7A, the Vimentin expression in mock group was significantly higher than all iC9-transduced groups (80%). Furthermore, a slight decrease in Vimentin expression was observed in iC9-transduced group after 300 nM AP20187 administration from 62.3% in non-treated to 57.6% in AP2018 treated group. The IHC results showed a low expression of E-cadherin in the mock group and a slightly increased expression in the iC9-transduced groups. In addition, immunoblotting was performed to further evaluate the expression of vimentin, E-cadherin, N-cadherin and Slug. As shown in Fig. 7B, the expression of vimentin, N-cadherin and slug was lower in the iC9-transduced groups than in the mock group, while the expression of E-cadherin increased.

### Caspase-9 activation reduces the migratory markers in MDA-MB231 cells

**Figure 8:**
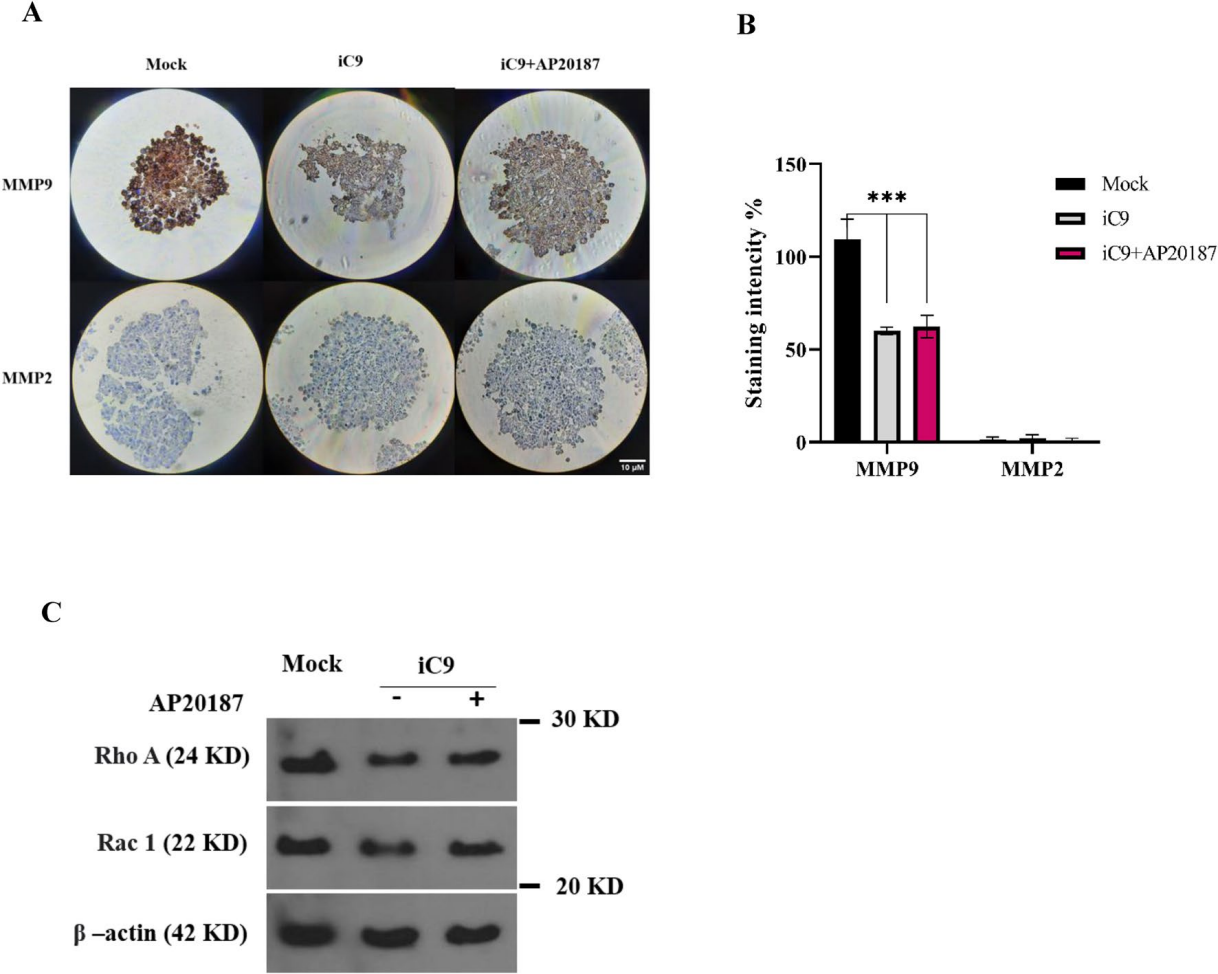
The migratory markers decrease upon AP20187 treatment. (**A**) IHC staining was performed to detect cellular MMP9, and MMP2 expression in the organotypic model. The positively stained cells were visualized as brown color (Magnification 40×, Scale bar is 10 μm). (**B**) The protein levels of Rho-A and Rac-1 in Mock, iC9-transduced cells with or without AP20187 treatment were assessed using immunoblotting. β-actin was considered as an internal control.

To elucidate the influence of the caspase-9 activation on metastatic MDA-MB-231 cells, the expression of metalloproteinase 2 (MMP2) and 9 (MMP9) within the organoid cells was assessed through IHC. As displayed in Fig. 8A,B the MMP9 expression in iC9-transduced cells were significantly decreased rather than mock-transduced cells (around 40%). Although, there was not observed significant differences between iC9-transduced groups. Furthermore, the IHC results did not show changes in MMP2 expression within the MDA-MB-231 cells. In addition, further evaluation of the migratory markers, Rho and Rac in Fig. 8C, revealed the reduced levels of the Rho A and Rac1 expression upon caspase-9 activation.

### Caspase-9 activation increased the sensitivity of MDA-MB231 cells to doxorubicin

**Figure 9:**
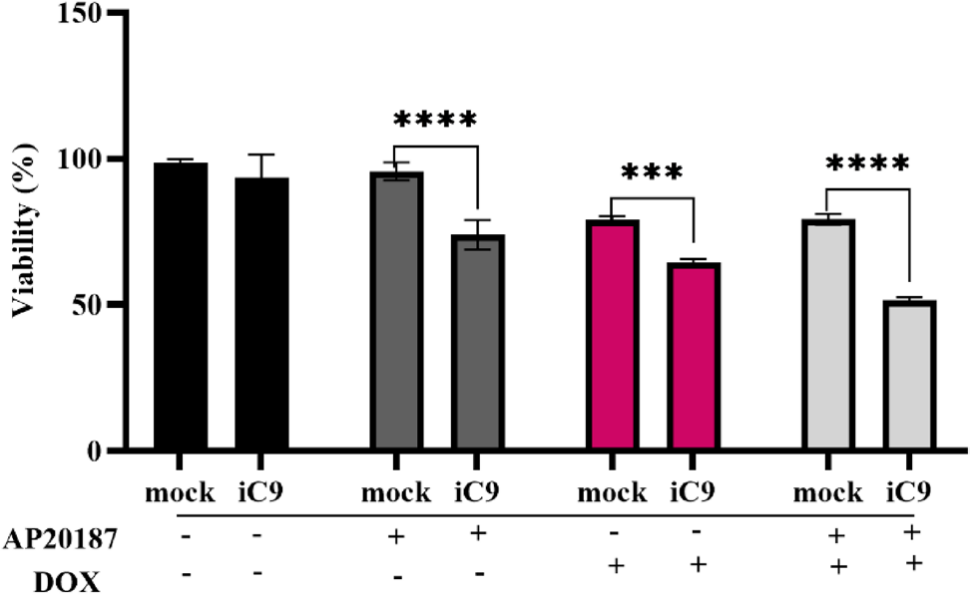
The iC9/AP20187 makes MDA-MB231 cells more sensitive to doxorubicin. The cytotoxicity of 0.5 μM doxorubicin on iC9-transduced cells with or without 300 nM AP20187 was examined using MTT assay. The data shown as mean ± SD for three replicates, and significant differences are denoted as; **p < 0.01, and ***p < 0.001.

To evaluate the impact of the iC9/AP20187 system on cellular sensitivity to doxorubicin, Mock and iC9-transduced cells were exposed to 300 nM AP20187, 0.5 μM Doxorubicin individually, or in combination over a 48-h period. As illustrated in Fig. 9, treatment with Doxorubicin led to a 19.5% and 29% reduction in cell viability in mock and iC9-transduced cells compared to their control groups, respectively. Notably, after AP20187 administration, the combination of these two treatments resulted in substantial 42.8% reduction in cell viability in iC9-transduced cells, however, reduction in cell viability in mock group was as same as Doxorubicin treatment result. Collectively, the iC9/AP20187 system could enhance the sensitivity of MDA-MB-231 cells to chemotherapy.

## Discussion

Metastasis in TNBC is a primary contributor to mortality in this malignancy. Most therapeutic interventions are effective in treating the primary disease but lose effectiveness when addressing the metastatic stage of TNBC. Hence, the development of innovative therapeutic approaches to tackle the metastasis of this cancer is imperative^5,27,28^.

Caspase-9 is a cysteine dependent aspartate driven protease primarily known as an apoptotic enzyme. Furthermore, recent research on Drosophila indicates a potential role of caspase-9 in the migration and invasion of cells^23,24^. This evidence implies the possible association of caspase-9 with metastatic behavior in breast cancer. Examining the linkage between clinical outcome and the expression of caspase-9 in breast cancer patients proves the association (Fig. 1). So that patients with lower level of caspase-9 shows adverse clinical outcome such as relapse and metastasis. Consequently, this study was conducted to examine the impact of caspase-9 on the growth characteristics, migration, and invasion behaviors of TNBC. To achieve this objective, a stable cell line expressing inducible caspase-9 was established. Subsequently, the increased expression and functionality of this variant within the cells were validated (Fig. 2). Furthermore, it was observed a modest basal activity in the absence of their activator (AP20187), a phenomenon previously documented in scientific literature^29^.

Following this, the cytotoxic effect of the iC9/AP20187 system on MDA-MB-231 cells was assessed (Fig. 3). Interestingly, the iC9-transduced cells treated with increasing concentrations of AP20187 did not exhibit a noteworthy level of cell death in the MTT assay. Only a 14.6% statistically significant decrease in absorbance was observed compared to the control which mightily interpreted as cell death. Likewise, the results obtained from flow cytometry did not reveal any discernible evidence of cell death. Consequently, it becomes evident that despite the activation of caspase-3 subsequent to the activation of caspase-9, the iC9/AP20187 system fails to induce substantial cell death in the MDA-MB-231 cell line. According to researches, various cell types exhibit distinct responses to inducible caspase-9-mediated cell death. For instance, iC9/AP20187 is unable to induce cell death in the human leukemic NB4 cells^30^. In contrast, MCF7 cells demonstrate heightened sensitivity to its cell death induction^31,32^. Moreover, research findings suggest substantial variability in the responses of distinct cell populations to apoptosis inducers, primarily associated with differences in initial iC9 expression and the XIAP/Caspase3 ratio^25^. Furthermore previous studies showed that AP20187 administration had not any cytotoxicity effects on human cells and tested in phase I clinical trial in healthy valentines and besides has not any known therapeutic effects^26,29,33^.

Moreover, the capacity to generate colony in iC9-transduced cells treated with AP20187 decreased significantly. Flow cytometry analysis of cell cycles revealed S-arrest in these cells, likely contributing to the reduced colony-forming ability. Several studies have demonstrated the participation of caspases in triggering the DNA damage response (DDR) during mitotic arrest. Mitotic stress triggers the non-apoptotic activation of classical caspases, which functions to preserve telomeres and cell viability.

2D models are suitable for initial studies, nevertheless, their incapacity to faithfully replicate cell-to-cell and cell-to-microenvironment interactions necessitates the development of more advanced models, such as 3D models, to address these limitations and more accurately mimic real physiological conditions^11–14^. Consequently, this study involved the development of an organotypic model for TNBC. The MDA-MB-231 cell line is renowned for its challenging nature in three-dimensional propagation^34^. On the other hand, fibroblasts, the predominant type of connective tissue cells in animals, play an active role in the formation and modification of the extracellular matrix. Studies have demonstrated that fibroblast cells induce the production of a stiffer extracellular matrix through the cross-linking of collagen, the most abundant protein in this matrix^35–37^. Consequently, in this research, human dermal fibroblast cells were used in co-culture with the MDA-MB-231 cell line (Fig. 4). The results indicated that MDA-MB-231 alone faced challenges in forming the solid organoids. Co-cultivation with fibroblast cells led to the development of rounder and more stable cell organoids. Furthermore, an increased proportion of fibroblasts resulted in greater compactness, with the 1:1 ratio exhibiting the highest retention of logarithmic phase and coherent structure compared to the other examined ratios. Sarah Mishriki, et al., developed a 3D co-culture model with different ratios of MDA-MB-231 and fibroblast cells using magnetic support. Their findings suggest that the presence of fibroblasts accelerates the formation of organoids, acting as a “glue” that holds three-dimensional cell structures together^38^. Furthermore, cells exhibiting high malignancy are widely recognized for their high mesenchymal and low epithelial characteristics, a trait that was confirmed by IHC results within the organotypic model^39,40^.

Following the generation and characterization of the organoids, their growth kinetic assessments upon caspase-9 activation displayed slower growth kinetics (Fig. 5). As previously mentioned, iC9 transduced cells underwent S-phase arrest following AP20187 treatment, significantly impeding the growth kinetics.

To evaluate the putative anti-metastatic effects of iC9/AP20187 system, migration and invasion assays were conducted. In these studies, Pan, a human monoclonal antibody EGFR antagonist, was employed as a reference anti-metastatic agent. It is indicated as a standalone therapeutic agent for metastatic colorectal cancer^41–43^. Moreover, given the overexpression of EGFR in TNBC, Pan is utilized either as a monotherapy or in combination with other therapeutic modalities for the treatment of this particular type of breast cancer^44,45^.

In all metastasis assays mock group showed a significant higher migration and invasion rate rather than all iC9-transduced groups. In scratch and Trans well assays, both AP20187 treated iC9-transduced cells and the Pan treated group exhibited reduced migration and invasion rates. Notably, the combination of AP20187 and Pan demonstrated an additional effect (Fig. 6, and see Supplementary Fig. S3 online). Likewise, organoid studies revealed that the iC9/AP20187 system effectively reduced cell migration and invasion. AP20187 exhibited a more potent inhibitory effect on invasion compared to Pan in the organoids. This difference can be attributed to the distinct nature and mechanisms of action of these molecules in the 3D environment, where EGF-R receptor expression varies which is not studied here^46^. Pan, an antibody, initiates downstream signaling by binding to EGF-R, while AP20187, a small molecule, and acts independently of the upstream signals and is less influenced by its microenvironment. In a study by Anna Gorelick-Ashkenazi, et al., it was demonstrated that minimal levels of caspase activity significantly impede migration in epithelial cells that have acquired invasive characteristics during development or ionization^24^. Furthermore, another study employing a sensitive marker for caspase-3 activity assess and live imaging techniques revealed the involvement of caspase-3 in the process of dorsal closure in Drosophila larvae, with its inhibition resulting in an accelerated closure process. In better words it was also observed that the activation of dronk leads to the generation of reactive oxygen species and can modulate the levels of myosin at the wound edges, consequently causing a delay in wound healing^23^.

Cellular cross-talk plays a positive role in metastasis development. Cells release chemokines that prepare other cells to recognize and exhibit metastatic behavior. Metastasis, however, is not a random process; it exhibits organotropism, meaning cells preferentially metastasize to specific organs. This phenomenon is influenced by various factors, including the host immune microenvironment, cancer subtypes, and molecular properties of cancer cells. In the context of breast cancer, cells preferentially metastasize to organs such as bones, brain, liver, and lungs^47^. In this study, bone marrow derived MSCs were selected to investigate the invasion (see Supplementary Fig. S3 online). The presence of mesenchymal stem cells was observed to increase the invasion rate of the mock-transduced cells compared to using media alone. Likely previous studies demonstrated that co-culturing human bone marrow MSCs with MDA-MB-231 cell line promoted the breast cancer metastasis^48,49^.

The EMT marks a pivotal event in tumor metastasis^50^, characterized by a shift from epithelial markers like E-cadherin to mesenchymal markers such as vimentin and N-cadherin^51^. E-cadherin, a calcium-dependent glycoprotein fostering adhesion among epithelial cells, contrasts with N-cadherin, absent in epithelial cells. Cadherin switching associates with heightened mobility and metastasis across various cancers, including breast cancer^52^. Vimentin, a type III intermediate filament cytoskeletal protein, emerges as another critical EMT marker and a regulator of mesenchymal cell migration^53–55^. Its overexpression in diverse epithelial cancers, including prostate, breast, lung, gastrointestinal, and central nervous system tumors, correlates with accelerated tumor progression and aggressive phenotypes^40,53,55–57^. Notably, recent research highlights the cleavage of Vimentin and downregulation upon caspase-9 activation in leukemic cells^58^.

Evaluation of Vimentin and N-cadherin expression reveals a significant reduction in the iC9-transduced group compared to mock cells, while E-cadherin expression notably increases. The transcription factor Slug, known to inhibit E-cadherin expression, also influences EMT dynamics^59^. Immunoblotting indicates a slight decrease in Slug expression during caspase activation (Fig. 7). This finding highlights the influence of the caspase-9 activation on the reduction of EMT in MDA-MB-231 cells.

Gelatinases, including MMP 2 and MMP 9, play crucial roles in tumor invasion, metastasis, tissue remodeling through the extracellular matrix, degradation of basement membranes, and promotion of angiogenesis^60,61^. It has been demonstrated that MMP 9, along with MMP 2, stimulates TGFβ signaling to support angiogenesis, tumor invasion, cell survival, and metastasis^62^. In our study, the IHC images indicated a noticeable reduction level of MMP9 expression from mock to iC9-transduced groups. Whereas, the MMP2 expression level was consist and almost negative in all groups (Fig. 8). A systematic analysis of MMPs expression in breast cancer tissue indicated that MMP 2 expression did not exhibit a significant up- or down-regulation compared to controls in grade II or III, while MMP 9 in cancer tissue, relative to control, was up-regulated in both grade II and III, with a more pronounced increase in grade III breast cancer^60^.

Furthermore, the Rho family of small GTPases plays a crucial role in restructuring the cytoskeleton, shaping cell morphology, and guiding cell migration. Among these, RhoA and Rac1 are prominent members whose irregular activity is linked to invasion and metastasis. Elevated levels of RhoA have been observed in lung, breast, and colon cancers. Additionally, a study demonstrated the indispensability of RhoA in the metastasis of MDA-MB-231 cells using a 3D model^63^. Moreover, research indicates that decreasing Rac1 expression can attenuate the migration and invasion of melanoma cells^64^. Immunoblotting analysis revealed decreased expression of both RhoA and Rac1 in iC9-transduced cells compared to the mock group (Fig. 8). This suggests that the activation of caspase 9 can impede migration and invasion in MDA-MB-231 cells by influencing EMT and reducing the expression of pivotal markers; RhoA, Rac1, and MMP9.

Furthermore, to assess the effectiveness of iC9/AP20187 in conjunction with chemotherapy techniques, we examined the cytotoxicity of 0.5 µM doxorubicin (Dox), the iC9/AP20187 system, or their combination. The iC9-transduced cells treated with AP20187 displayed heightened sensitivity to DOX in comparison to the mock group (Fig. 9), likely attributable to reduced proliferation and cell cycle arrest in the S-phase (Figs. 3, 5).

Altogether, our findings uncover the importance of caspase-9 activation in metastasis inhibition. However, this study provides a preliminary exploration of the anti-metastatic effects of caspase-9 in TNBC, emphasizing the need for further investigations into the involved signaling pathways and in vivo studies.

## Material and methods

### Cell culture

The human MDA-MB-231 cell line (IBRC C10684) and human Embryo kidney (IRBC C10139) were purchased from the Iranian Biological Resource Center, while the human primary Foreskin fibroblast cell (HFF) (BN_0012.1.32) and Bone marrow MSC(BM-MSC) (BN_0012.1.11) were obtained from BonYakhte Technology Co. The former three cell types were cultured in DMEM supplemented with 10% fetal bovine serum (FBS) and 1% penicillin–streptomycin, whereas the BM-MSC was cultivated in RPMI supplemented with 15% FBS and 1% penicillin–streptomycin. The cells were maintained at 37 °C in a 5% CO_2_ atmosphere, and the cell culture medium was refreshed every other day.

### Vector construction and lentiviral particle generation

The iC9 expressing Lentiviral vector (PCDH-iC9) was created by subcloning PCR-amplified iC9 from a synthetic PGH vector (Generay Biotech) into the Ecor1 and Xba1 restriction sites of a lentiviral pCDH-CMV-MCS-EF1-cGFP-T2A-pur (PCDH) plasmid. The cloning verification described in supporting information, Fig. S1. The primer sequences can be found in Table 1.

**Table 1:** The sequences of the applied primers for iC9 amplification.

Primers	Sequence (5′→3′)
Forward 1	ACCATGGGAGTGCAGGTGGAAACCATC
Forward 2	GTATGATCTAGAACCATGGGAGTGCAGGTG
Reverse	ATTGAATTCTTAGTCGAGTGCGTAGTCTGG

To produce complete lentiviral particles, HEK-293 cells were co-transfected with either the PCDH or the PCDH-iC9 vector, along with two packaging plasmids (pMD2.G and psPAX2), using PEI transfection reagent (Sigma). 48–72 h post-transfection, the supernatant containing lentiviral particles was collected and subsequently passed through a 0.45-μm filter. This supernatant is used immediately, and any remaining portion is stored at − 80 °C for future use.

### Establishment of a stable cell line

The MDA-MB-231 cells, pre-seeded at 50% confluence, were transduced with the aforementioned supernatants (containing lentiviral particles expressing either the PCDH or the PCDH-iC9 vector) in the presence of 8 μg/ml polybrene (Sigma) to enhance transduction efficiency. Subsequently, the media were replaced with fresh media after 24 h. Following this, the cells were subjected to expansion under Puromycin 1 mg/ml treatment for 30 days to select and maintain stably transduced cells, to validate the stable expression of iC9-PCDH (referred to as “iC9-transduced”) or PCDH vector (referred to as “mock-transduced”). The cells expressing green fluorescent protein (GFP^+^ cells) were visualized using fluorescent microscopy before quantifying the GFP expression via flow cytometry. Furthermore, the expression of iC9 at the RNA and protein levels was evaluated through real-time PCR and western blot analysis (Table 2).

**Table 2:** The sequences of the applied primers for RT-PCR.

Primers	Sequence (5′→3′)
Caspase9	
Forward	CATATCTAGTTTGCCCACACCC
Reverse	GCATTAGCGACCCTAAGCAG
GAPDH	
Forward	CATCAAGAAGGTGGTGAAGCAG
Reverse	AGCCAAATTCGTTGTCATACCAG

### MTT assay

Cells were seeded at a density of 1 × 10^4^ cells/well in each well of a 96-well plate. After 24 h, the iC9-transduced cells were treated with various concentrations of AP20187 (100–300 nM) and the mock-transduced cells treated with or without 300 Nm AP20187 in triplicate. Subsequently, the cells were incubated at 37 °C and 5% CO_2_ for 48 h. Following the incubation period, the medium was discarded, and 0.5 mg/ml Tetrazolium salt (3-(4, 5-dimethylthiazol-2-yl)-2, 5-diphenyltetrazolium bromide (MTT) diluted in PBS was added to each well and incubated for 4 h. Then, 100 μl DMSO was introduced to each well, and the absorbance was measured at 570 nm using an Elisa reader (BioTeK).

To assess the sensitivity of transduced cells to chemotherapy reagents, 1 × 10^4^ cells/well were seeded. The following day, the culture medium was replaced with a fresh one containing either 0.5 μM Doxorubicin (DOX) or 300 nM AP20187 alone, or a combination of both. A control group was included, which received no treatment. After 48 h, cell viability was assessed using the same protocol as previously described.

### Flow cytometry

In this study, flow cytometry was employed to assess the expression of GFP in transduced cells and also to evaluate the impact of AP20187 on the apoptosis and cell cycle in transduced cells.

To quantify the expression of GFP in both mock and iC9-transduced groups, a total of 50 × 10^4^ cells/well were seeded in a 6-well plate. After 24 h, the cells were analyzed using a flow cytometer (BD Accuri C6 plus).

For further investigation of apoptosis and cell cycle status, iC9-transduced cells were treated with or without 300 nM AP20187. At 48 h post-treatment, mock and iC9-transduced cells were harvested, washed with PBS, and stained by incubation in 100 μl of apoptosis buffer (1 μM PI and 0.5 μM calcein/AM in PBS) or cell cycle buffer (1 μM PI, 0.02% Ribonuclease A, 1% Triton X100) for 15 and 30 min, respectively, before assessment. The resulting data were analyzed using FlowJo software.

### Real-time PCR

The expression of iC9 in the established cell line was quantified 30 days post-transduction using Real-Time PCR (Applied Biosystems). Briefly, RNA extraction was performed using Bio Basic kit, and both the quality and quantity of the extracted RNA were measured using a spectrophotometer (Wilmington). Subsequently, 1 μg of RNA was utilized for cDNA-synthesis following the standard protocol of the AnaCell kit, and real-time PCR was conducted using High Rox™ RealQ Plus 2_ Master Mix Green (Amplicon).

The PCR process involved a 10 min initial denaturation, followed by 40 cycles of 5 s for denaturation, 30 s for annealing at 60 °C, and 30 s for extension at 72 °C.

### Western blotting

To investigate protein expression and the activation of caspase-9 in iC9-transduced cells, 50 × 10^4^ cells/well were seeded in each well of a 6-well plate. The following day, one group of iC9-transduced cells was exposed to 300 nM AP20187, while another group of iC9-transduced cells and a mock group were left untreated. After 48 h, cells were lysed using a RIPA lysis buffer and the total concentration of proteins was measured using the Bradford technique. The same amount of proteins was loaded and run onto a 12% polyacrylamide gel, which was subsequently transferred to a PVDF membrane. The membrane was blocked with a 2% nonfat dried milk solution in TBS-T buffer for 2 h. Following this, the membranes were exposed to relevant primary antibodies (β-Actin, caspase-9, caspase-3 (SANTA CRUZ, 1:1000)) for an overnight incubation at 4 °C. Subsequently, they were exposed to peroxidase-conjugated secondary antibodies (mouse anti-rabbit IgG-HRP (SANTA CRUZ, 1:1000)) for 2 h at room temperature. To visualize the protein bands, a chemiluminescence system with ECL advanced reagents was used, and their densities were analyzed using ImageJ software. The data was then normalized to the β-actin band intensities. Furthermore, to investigate the impact of iC9/AP20187 on the expression of EMT and migratory markers, the same protocol was employed. E-cadherin, slug, Rho, Rac 1, (SANTA CRUZ, 1:1000), Vimentin (e Bioscience, 1:1000) and poly clonal antibody N-cadherin (Elabscience, 1:1000) as primary antibodies and peroxidase-conjugated secondary antibody (mouse anti-rabbit IgG-HRP (SANTA CRUZ, 1:1000)) were used**.**

### Colony formation

To initiate colony formation, a total of 1 × 10^4^ cells/well mock and iC9-transduced cells were seeded in a 96-well plate. The following day, one iC9-transduced group was subjected to treatment with 300 nM AP20187, while the other groups remained untreated. Subsequently, the cells were maintained in a 37 °C, 5% CO_2_ environment for 48 h. Following this incubation period, the cells were trypsinized, and an equal number of 5000 cells were then seeded into each well of a 24-well plate. Two weeks later, the medium was aspirated, and the cells were fixed with methanol for 20 min, followed by a 30-min staining with Crystal Violet. Subsequently, the dye was removed, and the plate was washed. Finally, the colonies were photographed and quantified using ImageJ software.

### Spheroid formation and grow kinetic assay

To generate organoids, HFF and MDA-MB-231 cells were combined in five different ratios (1:1, 1:2, 2:1, 0:1) at a concentration of 0.2 × 10^4^ cells/well and cultured in agarose-coated 96-well plates. The growth kinetics of the organoids were assessed by monitoring their growth for 14 days, with photographs taken every other day using a light microscope (Zeiss). The alterations in their size were measured using ImageJ software.

Subsequently, to evaluate the impact of the iC9/AP20187 system on the growth kinetics of the organoids, the mock and iC9-transduced cells were combined with HFF cells at a total concentration of 0.2 × 10^4^ cells/well to generate the organoids. The following day, the iC9-transduced group was treated with 300 nM AP20187, while the other groups were left untreated. Organoids were photographed using a fluorescent microscope (Zeiss), continuing until the 13th day, and analyzed according to the mentioned protocol earlier.

### Scratch assay

In the wound healing assay, 10 × 10^4^ cells/well were seeded in each well of a 24-well plate. To prevent cells from proliferation during test, the culture media with reduced FBS was applied. The following day, once the cells had formed a consistent monolayer, a gentle scratch was created in the center of each using a sterile tip. The wells were then washed twice with PBS to remove any unattached cells. Thereafter, to prevent the proliferation effect of cells during test, the cells were cultured in 2% reduced FBS media supplemented both with and without the addition of AP20187. Additionally, a non-toxic concentration of Pan (50 μM) was used as demonstrated in Fig. S2, either independently or in combination with 300 nM AP20187. Imaging of the cells was conducted every day after 48 h using a fluorescent microscope (Zeiss). For analysis, the images were processed using ImageJ software, and the gap size was calculated. To determine the extent of cell migration inhibition for each group, the following formula was applied:

$${\text{Migration inhibition rate }}\% = \left( {\frac{{{\text{Gap width at }}48 {\text{ h}}}}{{\text{Initial gap width }}}} \right) \times 100$$

### 3D invasion assay

To conduct the invasion assay for organoids, a 4 mg/ml collagen solution was prepared by dissolving collagen powder in PBS and adjusting the pH to 7.4 with a specific amount of sodium bicarbonate solution. This mixture was kept on ice until use. Subsequently, 150 ml of the collagen solution was added to each well of 48-well plates and incubated overnight until the gel fully polymerized. The following day, any parts of the gel that had not solidified were removed, and the remaining gel was washed twice with PBS. After this, a 3-day spheroid was transferred to each well and allowed to attach to the surface of the gel for 20 min. Then, 100 ml of collagen solution was added, and the gel was allowed to polymerize for around 4 h before adding media supplemented with reduced 2% FBS, with or without 300 nM AP20187 or 50 μM Pan, or a combination of both. Subsequently, the organoids invaded for 3 days, and photographs were taken every 24 h using a fluorescent microscope (Zeiss). The invasion was quantified by counting the invaded cells using Image J software. The cell migration and invasion percentage were calculated by following formula:

$${\text{The cell migration and invasion }}\% = \left( {\frac{{\text{the number of invaded cells from oranoid to enviroment in h72}}}{{\text{ the number of invaded cells from oranoid to enviroment in h0 }}}} \right) \times 100$$

### Fluorescent imaging of organoids

To detect the MDA-MB-231 and HFF cells in organoids we used propidium iodide (PI) staining and GFP imaging. Briefly, the 3-day organoids were harvested and washed twice with PBS. Subsequently, they were fixed using a 4% paraformaldehyde solution before being stained with PI for 30 min under dark conditions. Following this, the spheroids were visualized using a fluorescent microscope with green and red filters (Olympus). The resulting images were then subjected to analysis and subsequently merged using ImageJ software.

### Histopathological analysis

To demonstrate that the spherical cell structures mentioned accurately mimic the key characteristics of a triple-negative tumor tissue, Hematoxylin and Eosin (H&E), and immunohistochemistry (IHC) staining was performed on Ki67, Vimentin, and E-cadherin. Briefly, the 3-day mock organoids were harvested and fixed in 4% paraformaldehyde before being dehydrated through a series of ethanol dilutions. Subsequently, they were embedded in paraffin and cut into 5 μm thick sections by a microtome. These sections were mounted on slides and rehydrated through a series of ethanol dilutions. Finally, they were stained H&E. To perform IHC, the Rabbit anti-human Ki67 monoclonal antibody (Clone SP6), Rabbit anti-human Vimentin monoclonal antibody (Clone SP20) and Mouse anti-human Cadherin E Monoclonal Antibody (Clone HECD-1) were applied. The detection was done using Master Polymer Plus Detection System (HRP) with DAB included, and the samples were counterstained with hematoxylins and mounted on slides. The slides were observed under a light microscope (Zeiss).

Furthermore, to investigate the impact of iC9/AP20187 on EMT and metastasis protein expression, the 3-day mock and iC9-transduced organoids were prepared one group of iC9-transduced organoids treated with 300 nM AP20187 for 48 h and subsequently utilized for IHC staining by using Rabbit anti-human Vimentin monoclonal antibody (Clone SP20), Mouse anti-human Cadherin E Monoclonal Antibody (Clone HECD-1), anti-human MMP9 monoclonal antibody and anti-human MMP2 monoclonal antibody.

### Association of caspase-9 with clinical outcome

Differential expression (DE) of caspase-9 and its association with breast cancer outcome were investigated for stratified patient cohorts using the publicly available Breast Cancer Integrative Platform (BCIP; http://www.omicsnet.org/bcancer/). DE was evaluated in 1337 TNBC patients and 5656 non-TNBC patients. Distant metastasis free survival (DMFS) and relapse free survival (RFS) analysis were performed in 1665 and 2619 breast cancer patients, respectively.

### Statistical analysis

Statistical analysis was conducted using Graph Pad Prism 8. *t*-tests, as well as one-way and two-way Analysis of Variance (ANOVA), were employed to determine statistically significant variances. Significance was established for p values < 0.05. Each experiment was independently replicated at least three times, and the mean is presented alongside the corresponding standard deviation (SD).

## Conclusion

The present study unravels the impact of caspase-9 on the metastatic behavior of a TNBC cell line in the monolayer or organotypic model. Contrary to inducing significant apoptosis, caspase-9 was found to reduce these cells' migratory and invasive behavior. Molecular investigations unveiled the decreased EMT and migratory markers in cells with active caspase-9/3/7, suggesting the observed anti-metastatic effects. Additionally, caspase-9 activation increased the sensitivity of TNBC cells to chemotherapy. The clinical outcome analysis also provides a proof-of-concept of the clinical value of caspase-9 expression in breast cancer patients. These findings offer caspase-9 could be a valuable therapeutic target in curing breast cancer**.** In this study, for the first time the positive role of caspase 9 in the metastatic behavior prevention of human breast cancer was reported however more studies needed to figure out the detail mechanisms behind this behavior.

### Supplementary Information

Supplementary Information.

## Data Availability

The datasets analysed during the current study are publicly available in the [Breast Cancer Integrative Platform (BCIP)] repository, [http://www.omicsnet.org/bcancer/].
