# Supplementary material for: Caspase-9 suppresses metastatic behavior of MDA-MB-231 cells in an adaptive organoid model
Source: Sci Rep. 2024 Jul 2;14:15116. doi: 10.1038/s41598-024-65711-z (PMC11219723; doi:10.1038/s41598-024-65711-z)
Supplement: Supplementary file 1 — Supplementary Information. [file 41598_2024_65711_MOESM1_ESM.docx]

**Supporting information**

**Title: Caspase-9 Mediated Inhibition of Metastasis: Insights from an Adaptive Organoid Model of Triple-negative breast cancer**

**The *iC9* gene was cloned in PCDH vector**

To verify the iC9 cloning gene in the PCDH vector, the PCDH-iC9 vector was digested by the BamHI restriction enzyme, which has one restriction site within the iC9 gene and another site on the PCDH vector. This enzymatic cleavage was expected to yield two fragments, measuring 8438 bp and 875 bp, respectively. Subsequently, gel electrophoresis was performed on the PCDH vector alone, PCDH-iC9, and PCDH-iC9 digested by BamHI. As illustrated in Fig. S1, all bands are positioned on the right sides, indicating the correct cloning of iC9 into the PCDH vector.


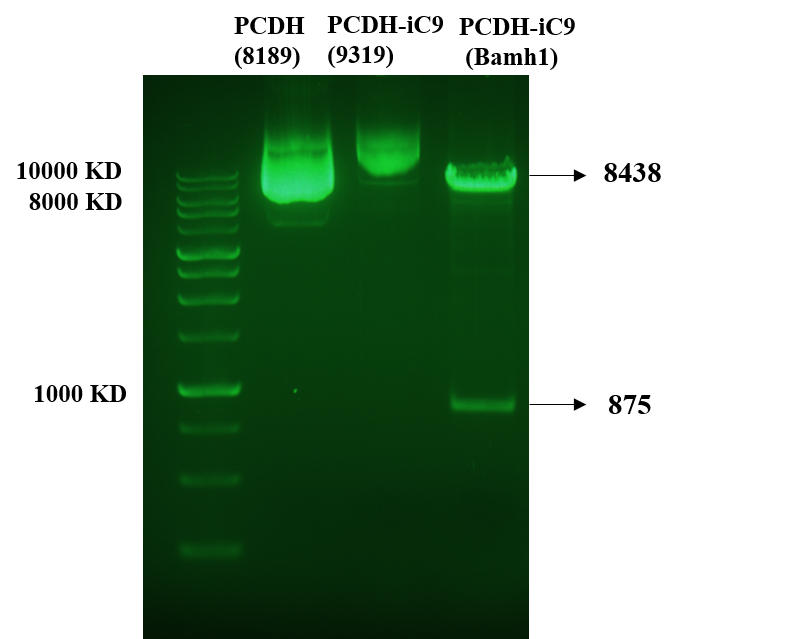


**Figure S1.** **Verification of iC9 cloning into PCDH Plasmid.** Agarose gel electrophoresis was performed on PCDH vector alone, PCDH vector containing iC9 gene (PCDH-iC9) and PCDH-iC9 vector digested by BamH1 restriction enzyme.

**Examining the cytotoxicity effect of Pan on MDA-MB-231 cell line**

In the investigation of the cytotoxic effects of Pan on the MDA-MB-231 cell line, an MTT assay was performed using increasing concentrations of Pan (50-250 μM) as previously described. The results presented in Fig. S2 demonstrated that Pan Treatment did not adversely affect MDA-MB-231 viability at concentrations below 150 μM, with only 7% and 26% cell death observed at 200 μM and 250 μM concentrations, respectively. Consequently, the 50 μM concentration was selected as the non-cytotoxic dose in this study.


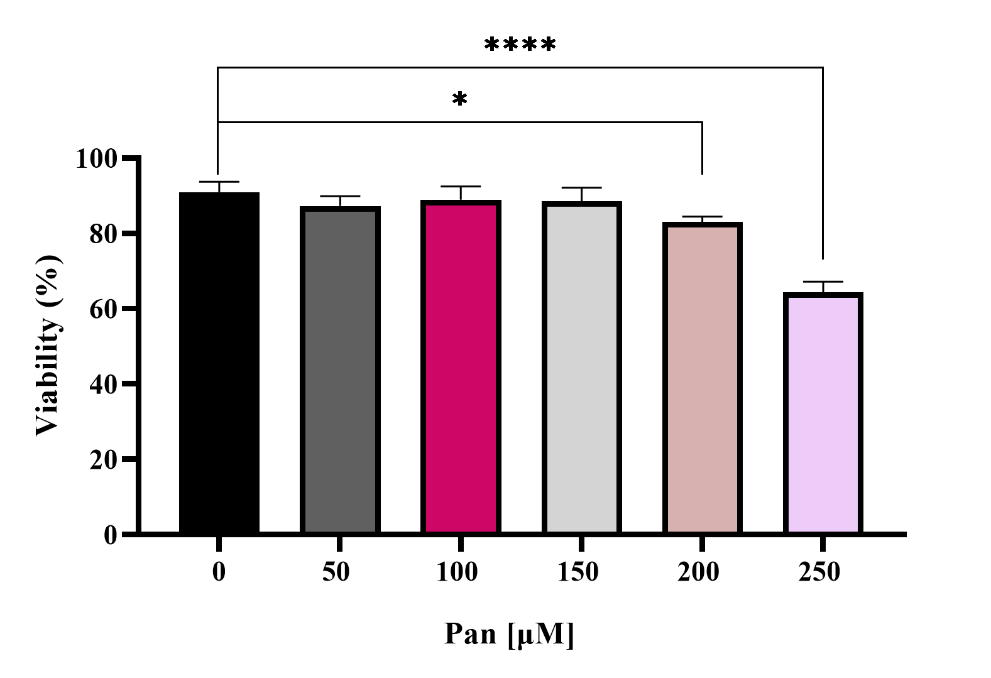


**Figure S2.** **The cytotoxicity effect of Pan on MDA-MB-231 cells.** The MTT assay was conducted to evaluate the viability of MDA-MB-231 cells following treatment with increasing concentrations of Pan (0-250 μM). The data are presented as the mean ± SD for three independent replicates, and the significance levels were indicated as * p < 0.05, and **** p < 0.001).

**Investigating the migration and invasion of MDA-MB-231 cells nearby MSCs upon caspase-9 activation**

To conduct the invasion assay, an 8μm pore size Transwell chamber was utilized. The wells were pre-coated with a 0.8mg/ml collagen solution obtained from bovine Achilles tendon, which was subsequently diluted in PBS and allowed to incubate for 2 hours to achieve complete gel polymerization. Any non-solidified gel was then rinsed away. In the upper chamber, 200μl of a cell suspension containing 0.5× 10^4 mock-transduced cells in FBS-free medium was seeded. For the lower chamber, 4×10^4 MSC_S_ were plated in RPMI with 15% FBS, or alternatively, the lower chamber was simply filled with 600μl of RPMI containing 15% FBS. In the case of iC9-transduced cells, the same number of cells in the upper chamber were suspended in a medium with or without 300 nM AP20187 or 50 μM Pan or combinations of these substances. In the lower chamber, 4×10^4 MSC_S_ were plated in RPMI supplemented with 15% FBS. Throughout the experiment, cells that migrated from the upper chamber membrane to the lower side were photographed in five different areas of the wells using a fluorescent microscope (Zeiss). Cell counts were manually performed using ImageJ software over a period of three days, and each sample was analyzed in triplicates.

The analysis revealed in Fig. S3A that the presence of MSC_S_ enhanced the invasion rate of mock-transduced MDA-MB-231 cells by 37.75%. Subsequently, all subsequent experiments were conducted in the presence of MSC_S_.

Fig. S3B demonstrated that both AP20187 and Pan Treatments significantly reduced the invasion of transduced cells compared to the non-treatment group (37.6% and 40.3%, respectively). Notably, the combination of AP20187 and Pan exhibited a synergistic effect in further reducing the invasion rate of the cells.

**
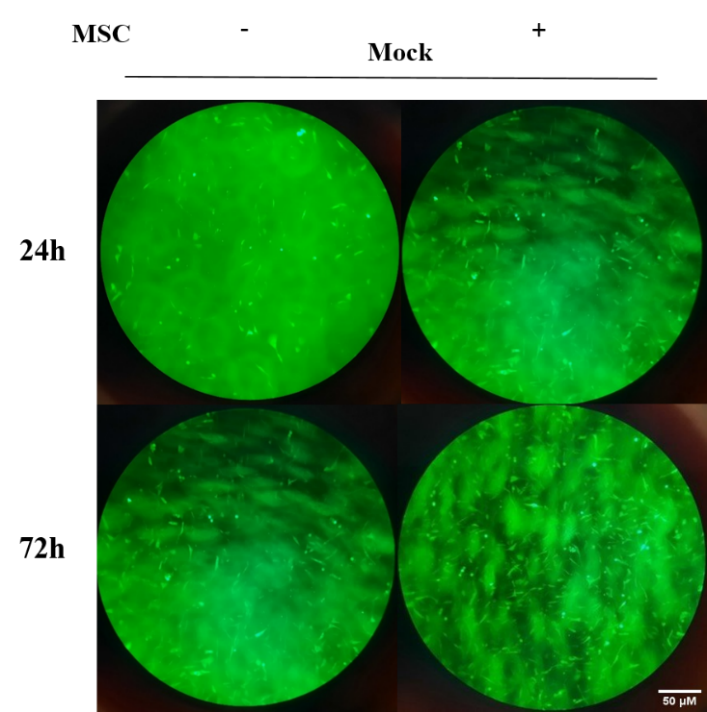
**

**A**

**
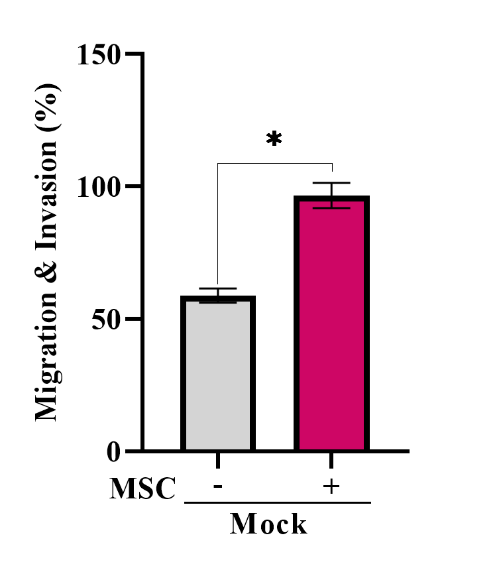
**

**
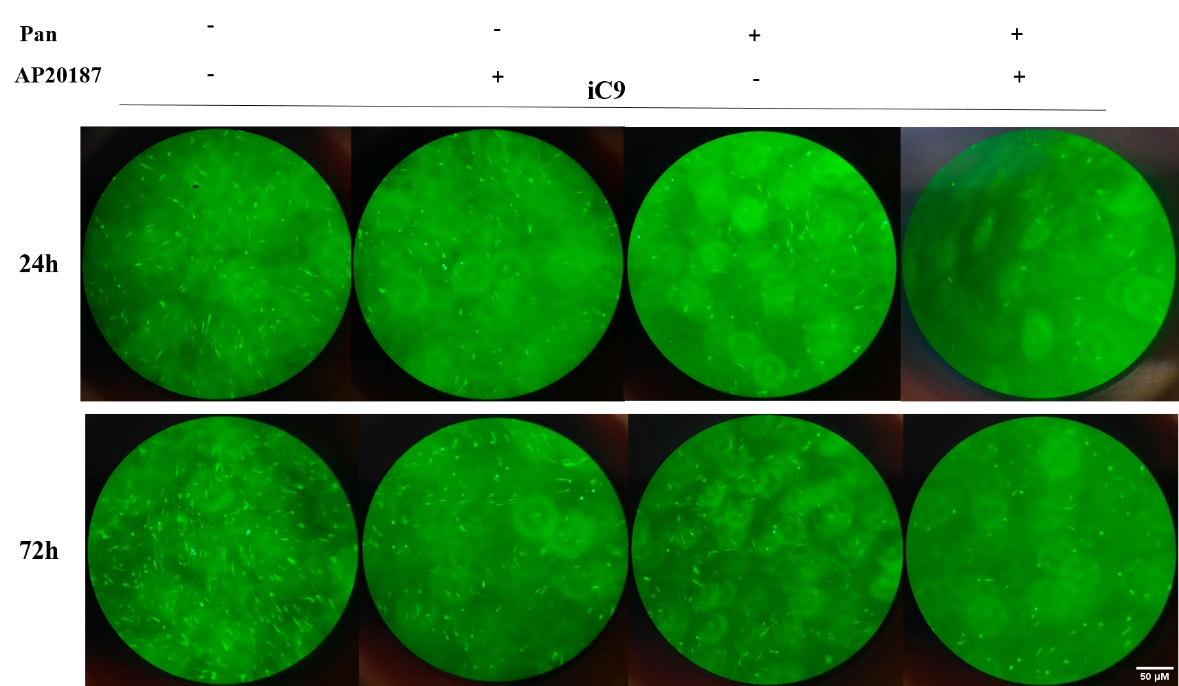
**

**B**

**
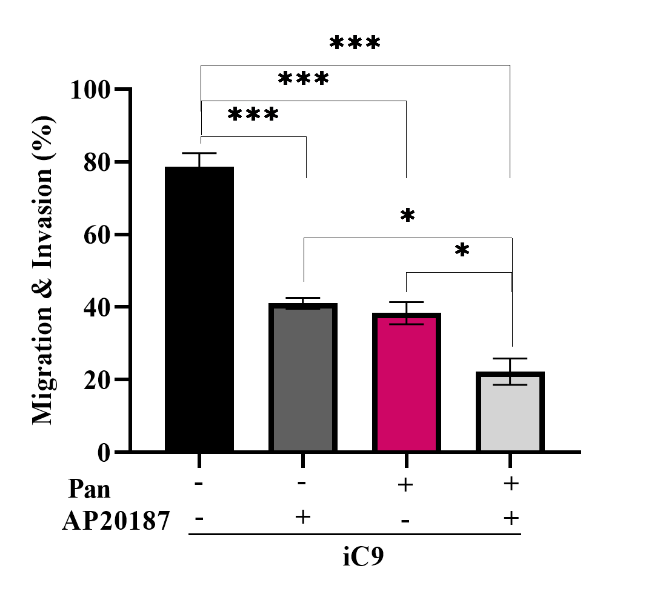
**

**Figure S3. The impact of MSCS co-culture on migration and invasion potential of transduced cells.** The fluorescent microscopy images and quantified invaded cells that passed through a collagen-coated membrane from the upper chamber to the bottom surface in trans well assay of Mock-transduced cells in the presence or absence of MSCS (A) and trans well assay of iC9-transduced cells in presence of MSCS under various treatment conditions, including with or without 300nm AP20187 and in combination with 50 μM Pan (B) The experiments were performed in triplicate and the results are presented as the mean of ± SD; * indicates P-value <0.05 and *** indicates p-value < 0.001. The Scale bar is 50μm.
